# The role of media in reducing and reinforcing stigma: A randomized controlled trial investigating the impact of positive and negative representations of visible difference

**DOI:** 10.1111/bjhp.70106

**Published:** 2026-08-21

**Authors:** Harley Teagle, Maia Thornton, Wylde M. C. Roberts‐Mills, Ella Guest

**Affiliations:** ^1^ Centre for Appearance Research University of the West of England Bristol UK; ^2^ School of Psychology University of Plymouth Plymouth UK

**Keywords:** appearance diversity, media representations, positive body image, stigma, visible difference

## Abstract

**Objectives:**

Individuals with visible differences often experience appearance‐related stigma and discrimination, reinforced by negative media portrayals. In contrast, positive portrayals may challenge stereotypes and promote acceptance. This study examined whether exposure to positive, negative or neutral images of visible difference influences appearance‐related stigma, body appreciation and broad conceptualizations of beauty. It was hypothesized that positive images would decrease stigma and increase body appreciation and broad conceptualizations of beauty, whereas negative images would increase stigma and reduce broad conceptualizations of beauty.

**Design:**

An online randomized controlled experiment using a mixed repeated‐measures design compared three conditions (positive, negative, neutral) across pre‐ and post‐exposure.

**Methods:**

A sample of 103 adults viewed 10 images of individuals with visible differences presented in one of three conditions: positive (positive captions), negative (villains with visible differences and negative captions) or neutral (without captions). Participants completed pre‐ and post‐measures of appearance‐related stigma, body appreciation and broad conceptualizations of beauty. Repeated‐measures ANOVAs examined within‐ and between‐group changes.

**Results:**

Appearance‐related stigma significantly increased in the negative condition, while remaining unchanged in the positive and neutral groups. Body appreciation significantly increased from pre‐ to post‐ across all conditions. No significant effects emerged for broad conceptualizations of beauty.

**Conclusions:**

Negative portrayals of visible difference may reinforce stigma, highlighting the need to discourage such depictions in media. While positive exposure did not significantly reduce stigma, viewing images of visible difference increased observers' body appreciation, indicating potential downward social comparison. Future research should explore strategies to strengthen stigma reduction and broaden conceptualizations of beauty.

**Statement of Contribution**

**What is already known on this subject?**

- Individuals with congenital or acquired visible differences are often subject to staring, intrusive questioning, and disadvantage in educational and occupational contexts (Rumsey & Harcourt, 2012; Stone & Wright, 2013; Stevenage & McKay, 1999).
- Negative representations in film and television have historically reinforced harmful stereotypes, whereas emerging evidence suggests that positive or body‐positive media portrayals may improve attitudes towards people with visible differences (Croley et al., 2017; Kilby & Mickelson, 2025; Mathews et al., 2023; Smith, 2012; Stein et al., 2024).

**What does this study add?**

- Negative portrayals of visible difference significantly increase appearance‐related stigma.
- Viewing images of visible difference increases observers' body appreciation.
- Positive portrayals alone may be insufficient to significantly reduce stigma.

## INTRODUCTION

Interest in appearance has never been more prevalent, with an increasing number of people aspiring to meet unrealistic and narrow sociocultural ideals (e.g., thin but toned bodies, clear skin, Grogan & Masterson, 2012). Research has suggested that beauty is both rewarding and rewarded (Datta Gupta et al., 2016). For example, individuals considered ‘attractive’ by society are perceived to be more competent, socially skilled, hireable, sexually experienced and, overall, more content and satisfied with life. These appearance ideals are becoming more extreme: Women are expected to have long slim legs, clear, flawless skin, big eyes and a flat stomach (Schaefer et al., 2019) and men are expected to be muscular, have a shaped torso and low body fat, as well as having a six pack and straight, white teeth (Kling et al., 2016). Especially in Westernized nations, there is a pervasive focus on being physically attractive and the belief that ‘what is beautiful is good’ (a term coined by Dion et al., 1972), which can be detrimental to those who fall outside of this ‘norm’, including individuals living with a visible difference, who frequently experience negative reactions to their appearance and societal marginalization, driven by social stigma (Harcourt et al., 2025).

Approximately one‐fifth of the UK population are estimated to have a visible difference, with 1.3 million of those estimated to have a significant visible difference (Changing Faces, 2019). These statistics include those with any condition, injury or treatment side effect that results in an appearance generally considered by society to be ‘different from the norm’ (Rumsey & Harcourt, 2012). Furthermore, these visible differences can either be present at birth (congenital; e.g., cleft lip and/or palate, birthmarks), acquired through trauma (e.g., burn injuries, scarring), surgical intervention (e.g., mastectomy) or disease (e.g., alopecia, psoriasis; Rumsey & Harcourt, 2004). Nevertheless, individuals living with congenital or acquired visible differences can face profound psychosocial challenges. Staring, unwanted questioning, audible comments, and aversive and avoidant behaviours are often displayed by the public (Mantey, 2017; Rumsey & Harcourt, 2012; Sarwer et al., 2022). Stigmatization of people with visible differences also extends into educational and professional settings: with a report by Changing Faces (2021) finding that children with visible differences are twice as likely to experience bullying at school than those without visible differences and research by Stone and Wright (2013) displaying that employers are less likely to invite applicants who disclose a visible difference to interview.

Stigmatization is defined by Pescosolido and Martin (2015) as a social process that devalues a target through the conferring of labels and stereotyping, resulting in social exclusion and discrimination. A recent review of the literature by Rasset et al. (2022) applied the social stigma framework conceptualized by Pryor et al. (2012) to examine stigma towards facial difference, with implications for understanding visible difference stigma more broadly. The review identified abundant research evidencing public stigma, in the form of affective, cognitive and behavioural reactions towards people with facial differences, with a potential significant impact on their lives and well‐being. Furthermore, many of the included studies display the importance of structural stigma (i.e., the way society confirms and legitimizes stigma) in perpetuating the stigmatization of people with facial difference through the lack of positive representation and prevalent negative representations within the media (e.g., within film and television). Indeed, within film, people with visible differences are often portrayed as emotionally disturbed and sinister villains who pose a threat to society (Croley et al., 2017; Smith, 2012), for example in characters such as the Phantom of the Opera and Freddy Krueger, and more recently in several villains in the latest instalments of the James Bond film series, as well as the Grand High Witch in the 2020 film adaptation of Roald Dahl's *The Witches*. The defining characteristics of these characters are their visible differences and their villainous behaviour, which reinforces unfavourable societal stereotypes, with a lasting impact on how people with visible differences are treated within society (Harcourt et al., 2025; Stock et al., 2013; Swift & Bogart, 2021).

Despite this, emerging research suggests that exposure to positive media representations can improve perceptions of people with visible differences. For instance, Mathews et al. (2023) found greater positivity in evaluations and reduced stigmatizing attitudes towards people with visible differences post‐exposure to the Guardian's documentary ‘Scars’ which depicts the lived experiences of individuals with scarring, demonstrating the potential for positive media representation to challenge negative societal perceptions. Furthermore, social media is increasingly being explored as a tool for changing perceptions of people with visible differences and reducing stigma (Guest et al., 2025). Recent years have seen a proliferation of ‘body positive’ content on social media, which aims to challenge narrow appearance ideals and represent a diverse array of bodies of different shapes, sizes, colours, features and abilities, with the aim of fostering body acceptance, appreciation and broad conceptualizations of beauty (Cwynar‐Horta, 2016). Observing these body‐positive images has been found to increase psychological, social and emotional well‐being, including positive mood and both satisfaction and appreciation of one's body, but it also significantly reduces the stigma towards diverse bodies (Kilby & Mickelson, 2025; Stein et al., 2024). Exposure to social media content depicting a diverse range of appearances, such as individuals with physical disabilities or visible differences, when framed in a body‐positive manner can also improve observers' perceptions of their own appearance by broadening conceptualizations of beauty and shifting standards of what is considered beautiful or attractive (Cohen et al., 2019; Dedecker et al., 2025). As such, by broadening narrow conceptualizations of beauty, positive representations of diverse appearances are proposed to serve the dual purpose of outwardly reducing appearance‐related stigma and inwardly improving one's own body appreciation. However, it is unclear whether exposure to imagery including individuals with visible differences can alone change attitudes, as an unpublished pilot reported by Guest et al. (under review) found that exposure to Instagram posts including photos of people with visible differences with no accompanying information or context was insufficient to significantly alter stigma. Thus, the inclusion of a positive framing and an educational or contextual caption may be critical to alter broad conceptualizations of beauty and subsequently reduce stigma.

The stigma‐reducing effects of viewing such body‐positive content may be understood through the lens of Parasocial Contact Hypothesis (Schiappa et al., 2005), which proposes that positive exposure to members of a stigmatized outgroup through media can build one‐sided relationships which mirror direct intergroup contact, reducing prejudice and increasing positive perceptions in ways comparable to a real‐world interaction (Bond, 2021; Ray & Husnu, 2025). This process operates through fostering identification with the outgroup, reducing perceived social distance and unpredictability and enabling perspective taking; however, its effectiveness is contingent upon the representations to which individuals are exposed being of a positive nature (Schiappa, 2024). Accordingly, parasocial contact offers a plausible mechanism to explain how body‐positive social media content has been shown to reduce prejudicial attitudes towards diverse bodies (e.g., Kilby & Mickelson, 2025). However, as much of the existing research on body positivity has focused primarily on obesity stigma (Flint, 2015), it remains unclear whether positive representations of people with visible differences can effectively reduce stigmatizing attitudes. As such, further exploration is needed to assess whether exposure to positive portrayals of people with visible differences can enact parasocial contact and influence stigma.

Overall, although a plethora of research showcases how negative attitudes towards visible difference occur, there is a lack of research exploring ways to promote acceptance and reduce stigma towards individuals who are visibly different (Harcourt et al., 2025). Therefore, based on the limitations of previous research exploring appearance‐related stigma, the present study was conducted to address these issues. An online randomized controlled experimental pre–post‐design was used to investigate whether exposure to images of visible differences can impact individuals' levels of stigma towards visible difference, and whether exposure leads to changes in the viewers' body appreciation and broad conceptualizations of beauty. This study is important because appearance‐related stigma has been found to cause significant psychosocial implications for individuals living with a visible difference (Rumsey & Harcourt, 2004). Furthermore, unlike previous research, which has aimed to solely assess the influence of exposure to positive or negative representations on stigma, this study aimed to do both. This was achieved by showing respondents images of visible difference displayed within a positive, negative or neutral context *(see experimental stimuli)*.

It was hypothesized that from pre‐ to post‐test this experimental exposure would:

- significantly decrease appearance‐related stigma for participants in the positive condition, and significantly increase stigma for participants in the negative condition, relative to the neutral condition.
- significantly increase body appreciation for participants in the positive condition, to a significantly greater extent than any change observed in the negative and neutral conditions.
- significantly increase broad conceptualizations of beauty for participants in the positive condition yet significantly decrease broad conceptualizations of beauty for participants in the negative condition, relative to the neutral condition.

## METHOD

### Participants

The participants consisted of undergraduate students, aged 18 years and above, recruited from the undergraduate psychology Participant Pool at University of the West of England (UWE), as well as participants recruited externally either via social media or via snowball sampling through word of mouth. Prior to recruitment, the study was approved by the UWE Psychology Ethics Committee, and British Psychological Society ethical guidelines were followed. Furthermore, all participants who completed the experiment via the Participant Pool were compensated by receiving a course credit.

A total of 123 responses were collected. However, 16 responses in which participants had failed to complete the second timepoint, 3 responses in which the participants had failed to complete either timepoint, and 1 duplicate response were removed leaving a final sample of *N* = 103 participants. A G*Power post‐hoc sensitivity analysis indicated that the final sample size (*N* = 103) provided 95% power (*α* = 0.05) to detect a small‐to‐moderate effect (*f* = 0.20) within a mixed‐effects design. Participants were randomly assigned to one of the three conditions—positive exposure, negative exposure or neutral exposure. In total, there were 34 participants within the negative condition, 34 within the positive condition and 35 within the neutral group. For a breakdown of sample characteristics and demographic information, see Table 1.

**TABLE 1:** Sample characteristics and demographics by condition group.

Characteristic	Total (*N* = 103)	Condition		
		Negative (*n* = 34)	Positive (*n* = 34)	Neutral (*n* = 35)
Age, *M* (*SD*)	22 (7.95)	21.90 (7.47)	22.10 (8.57)	22 (7.98)
Gender, *n* (%)				
Female	73 (70.9%)	23 (67.6%)	22 (64.7%)	28 (80%)
Male	27 (26.2%)	11 (32.4%)	10 (29.4%)	6 (17.1%)
Non‐binary / third gender	2 (1.9%)	–	1 (2.9%)	1 (2.9%)
Prefer not to say	1 (1%)	–	1 (2.9%)	–
Sexuality, *n* (%)				
Heterosexual	79 (76.7%)	29 (85.3%)	28 (82.4%)	22 (62.9%)
Gay/lesbian	3 (2.9%)	1 (2.9%)	1 (2.9%)	1 (2.9%)
Bisexual	16 (15.5%)	3 (8.8%)	2 (5.9%)	11 (31.4%)
Pansexual	2 (1.9%)	–	1 (2.9%)	1 (2.9%)
Prefer not to say	3 (2.9%)	1 (2.9%)	1 (2.9%)	–
Ethnicity, *n* (%)				
White	80 (77.7%)	29 (85.3%)	26 (76.5%)	25 (71.4%)
Arab	2 (1.9%)	1 (2.9%)	–	1 (2.9%)
Asian	11 (10.7%)	2 (5.9%)	4 (11.8%)	5 (14.3%)
Mixed and multiple ethnicities	8 (7.8%)	2 (5.9%)	2 (5.9%)	4 (11.4%)
Prefer not to say	2 (1.9%)	–	2 (5.9%)	–
Participant visible difference status, *n* (%)				
Yes	13 (12.6%)	3 (8.8%)	4 (11.8%)	6 (17.1%)
No	82 (79.6%)	29 (85.3%)	27 (79.4%)	26 (74.3%)
Don't know	7 (6.8%)	2 (5.9%)	2 (5.9%)	3 (8.6%)
Prefer not to say	1 (1%)	–	1 (2.9%)	–
Knows someone with a visible difference, *n* (%)				
Yes	69 (67%)	23 (67.6%)	20 (58.8%)	26 (74.3%)
No	28 (27.2%)	11 (32.4%)	11 (32.4%)	6 (17.1%)
Don't know	6 (5.8%)	–	3 (8.8%)	3 (8.6%)
Friends with someone with a visible difference, *n* (%)				
Yes	56 (54.4%)	16 (47.1%)	17 (50%)	23 (65.7%)
No	45 (43.7%)	18 (52.9%)	17 (50%)	10 (28.6%)
Prefer not to say	2 (1.9%)	–	–	2 (5.7%)
Follows someone with a visible difference on social media, *n* (%)				
Yes	79 (76.7%)	26 (76.5%)	27 (79.4%)	26 (74.3%)
No	22 (21.4%)	8 (23.5%)	7 (20.6%)	7 (20%)
Prefer not to say	2 (1.9%)	–	–	2 (5.7%)

### Materials

#### Visible difference specific questions

To assess respondents' exposure to, and knowledge regarding visible difference prior to the study, several multiple‐choice questions were utilized. These asked respondents whether they considered themselves to have a visible difference, whether they knew somebody with a visible difference, whether they were friends with someone with a visible difference, as well as whether they follow anyone on social media with a visible difference (with each question being answered with either ‘yes’, ‘no’ or ‘do not know’).

### Outcome measures

*Appearance‐related stigma* was measured using an adapted version (Guest et al., under review) of the Perceived Stigmatisation Questionnaire (PSQ; Lawrence et al., 2006) to measure self‐reported stigma towards those living with a visible difference. Participants completed the measure pre‐ and post‐experimental exposure. The original PSQ examines the extent to which an individual perceives others are exhibiting stigmatizing behaviours towards them; however, it was adapted to assess the extent to which members of the general population feel they are stigmatizing individuals with visible differences. The adapted scale contained 14 items questioning respondents on how they would act around someone with a visible difference (e.g., ‘I would stare at them’, ‘I would be kind to them’). The scale is completed using a 5‐point Likert scale (*1 = Never*, *5 = Always*) and is scored by calculating the mean score (questions 9–14 are reverse coded). Higher item scores indicate greater perception of stigmatizing behaviours. Despite the PSQ having been shown to have good internal consistency indices and convergent and discriminant validity (Lawrence et al., 2006), this adapted version has not been validated but has been used in other studies (Guest et al., under review). Cronbach's alphas for the current study were .77 and .91 at Time 1 and Time 2, respectively.

*Broad conceptualization of beauty*, which is the extent individuals define beauty widely within external and internal characteristics, was measured using the Broad Conceptualisation of Beauty scale (BCBS; adapted from Tylka & Iannantuono, 2016) at pre‐ and post‐experiment. The authors adapted the items to be gender neutral (e.g., woman was changed to person, and beauty was changed to attractiveness). The scale is comprised of 7 items, each item asking respondents in which way they feel beauty is defined (e.g., ‘A person's confidence level can change my perception of their physical attractiveness’). Responses are anchored on a 7‐point Likert scale (*1 = Strongly Disagree, 7 = Strongly Agree*); scores on the items are averaged. Higher scores indicate a broader conceptualization of beauty. Again, this adapted version has not been validated, but the original measure has good evidence of psychometric validity (Tylka & Iannantuono, 2016). Cronbach's alphas for the current study were .73 and .84 for Time 1 and Time 2, respectively.

*Body appreciation*, which measures individuals' acceptance of favourable opinions towards, and respect for their bodies, was assessed using the State Body Appreciation Scale (SBAS, Homan, 2016) pre‐ and post‐experiment. Ten statements asking respondents how they think and feel about their body in the current moment (e.g., ‘I feel love for my body’, ‘I respect my body’) are rated on a 5‐point Likert scale (*1 = Never, 5 = Always*), with scores averaged to obtain an overall body appreciation score. Higher scores reflect greater body appreciation. The SBAS has evidence of psychometric validity and reliability (Homan, 2016). Cronbach's alphas for the current study were .95 and .96 at Time 1 and Time 2, respectively.

### Experimental stimuli

The experimental stimuli included a total of 30 images of individuals living with a variety of visible differences. Depending on the condition participants were randomly allocated to (either positive, negative or the neutral group), different images were shown. Each condition consisted of 10 images, with each image being shown on screen for 10 s before allowing participants to move on. This ensured participants had enough time to look at the photograph, as well as being able to read the corresponding captions (described below). All images were sourced either from social media platforms such as Instagram, the Changing Faces website, or from internet search engines. Due to copyright restrictions, the images are not included within the manuscript.

To confirm the images were appropriate for each condition, a pilot study was conducted (*N* = 26), whereby participants were shown the entire stimulus set in a random order and were asked to identify which condition they thought the image belonged to, and their confidence in the categorization (using a visual analogue scale (VAS) ranging from 0 to 100). Means and standard deviations for each photograph were then analysed to determine whether the images were appropriate for their assigned experimental condition. Photographs with mean values of 70 and above were retained, with 12 images being removed.

#### Positive condition

These images portrayed visible difference in a positive light. This was achieved by showing images of individuals either smiling, laughing or proudly showing off their condition. These images also contained corresponding captions derived from posts from visible difference influencer social media accounts. The captions included descriptions of the individual's condition and life, how their condition has affected them, as well as how they acquired their condition (e.g., ‘After I was severely burned in an accident, my life turned upside down. I fought for my life and my future. Now, I'm 32 and have learnt to love the skin I'm in, embracing being a burn survivor, and seeing every scar as a step in a long journey’ (details altered to maintain source anonymity)).

#### Negative condition

These images portrayed visible difference negatively. This was achieved by using images of well‐known movie villains with visible differences, such as the Joker and Voldemort. These images also had corresponding captions, providing a description of the villain, alongside all the crimes they had committed (e.g., ‘The joker is a supervillain, who is portrayed as a criminal mastermind. This psychopathic character is responsible for brutally murdering hundreds of innocent individuals, one of which being his own mother’).

#### Neutral condition

Images within the neutral condition included photographs of real people with different visible differences, framed in a neutral way. In images including an individual's face, a neutral facial expression was maintained signifying neither a positive nor negative emotional state. To further maintain neutrality, these images had no corresponding captions attached to them.

### Procedure

Recruited participants were first directed to the online survey platform Qualtrics via a link where they read through an information sheet explaining aspects of the study and how their results will be used, before providing informed consent. Demographic information was then collected, followed by specific questions regarding visible difference and the outcome measures (adapted PSQ, adapted BCBS and SBAS). Following this, participants were randomly allocated into one of three conditions by Qualtrics (either positive, negative or the neutral group), where they were shown 10 images of individuals with a variety of visible differences (including birthmarks, vitiligo, burn scars and alopecia) with each image being shown for 10 s before allowing participants to move on. Depending on which condition participants were allocated to determined which images they saw (*see Experimental Stimuli*). After viewing all 10 images, participants were then asked to complete the outcome measures again. After completion, all participants were then presented with a debrief form outlining the true aims of the study, as initially participants were solely told the purpose of the study was to explore how people view and perceive images of people living with a visible difference.

### Data analysis

A total of 16% of cases had missing data across the primary variables. To evaluate the pattern of missingness, Little's Missing Completely at Random (MCAR) test was conducted. The result was non‐significant [*X* ^*2*^ = 32.27, *df* = 31, *p* = .404] suggesting that data were missing completely at random. As the MCAR assumption was met and the post‐hoc sensitivity analysis indicated that the final complete case sample retained sufficient statistical power (see ‘*participants*’ for details), a complete case analysis was conducted. A series of 3 (group) × 2 (time) repeated‐measures mixed‐effects ANOVAs were carried out to examine group differences across time for each of the outcome measures. Significant results were followed up with post‐hoc Bonferroni‐corrected simple main effects analyses.

## RESULTS

### Preliminary analysis

Preliminary analyses were administered to assess the reliability of the measures, to identify any outliers and examine normality. For each outcome variable and timepoint, the skewness (ranging between −1.17 and 1.50) and excess kurtosis values (ranging between 0.07 and 2.58) fell within an acceptable range and did not exceed rule of thumb ranges for severe non‐normality (±2 for skewness and ±4 for excess kurtosis; Hair et al., 2010; Kline, 2011). Moreover, ANOVAs are robust against violations of the normality assumption, meaning the type I error rate remains close to the alpha level specified in the test (Schmider et al., 2010). ANOVAs were also run to see whether the groups were comparable at baseline. The results revealed the groups were comparable, highlighted by there being no significant differences regarding for appearance‐related stigma [*F*(2, 100) = .686, *p* = .506], broad conceptualizations of beauty [*F*(2, 100) = 2.110, *p* = .126] or body appreciation [*F*(2, 100) = .321, *p* = .803]. Mean and standard deviations for the outcome measures by group at each time point is presented in Table 2.

**TABLE 2:** Means and standard deviations for outcome measures at each Time 1 and Time 2.

Outcome	Possible rang of score	Positive condition (*M* (*SD*))		Negative condition (*M* (*SD*))		Neutral condition (*M* (*SD*))	
		Time 1 (*n = 34*)	Time 2 (*n = 34*)	Time 1 (*n =ȉ34*)	Time 2 (*n = 34*)	Time 1 (*n = 35*)	Time 2 (*n = 35*)
Perceived stigma	1–5	1.45 (0.30)	1.35 (0.37)	1.54 (0.37)	1.92 (0.78)	1.52 (0.37)	1.62 (0.57)
Body appreciation	1–5	3.35 (0.79)	3.55 (0.92)	3.29 (0.82)	3.36 (0.78)	3.21 (0.95)	3.33 (0.95)
Broad conceptualization of beauty	1–7	6.00 (0.79)	6.21 (0.80)	6.07 (0.79)	6.10 (0.74)	6.34 (0.57)	6.25 (0.79)

### Appearance‐related stigma

For stigma, there were significant main effects of Group (positive vs. negative vs. neutral) [*F*(2, 100) = 5.24, *p* = .007, η^2^ = .095] and Time (Pre‐experiment vs. Post‐experiment) [*F*(1, 100) = 7.69, *p* = .007, η^2^ = .071] and a statistically significant interaction effect [*F*(2, 100) = 8.52, *p* = <.001, η^2^ = .146].

**FIGURE 1:**
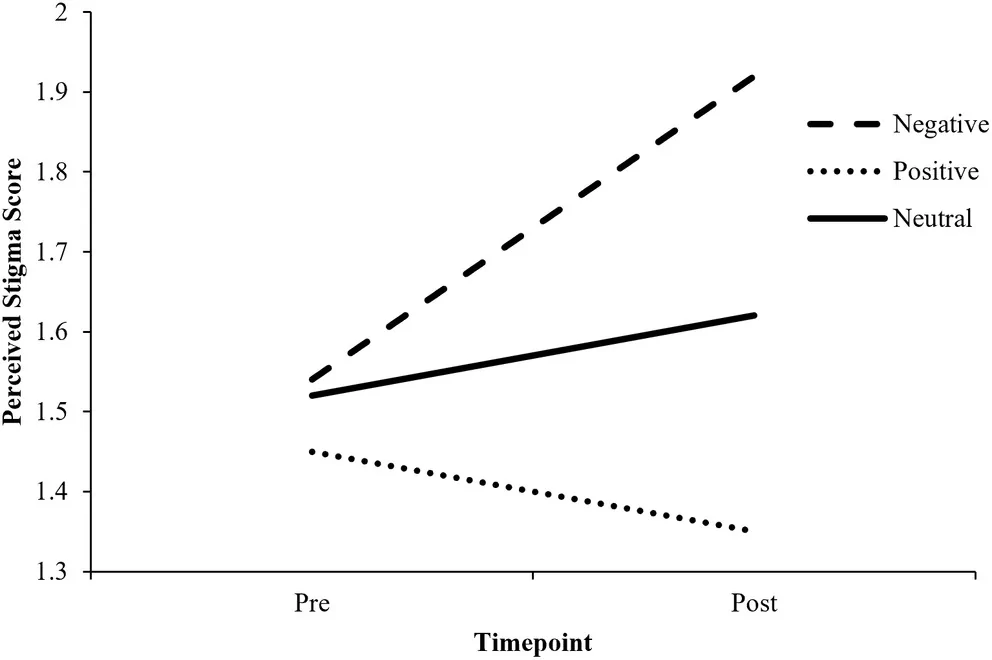
Changes in PSQ scores as a function of timepoint and condition group. Descriptive Plots showing the effects of Time on mean PSQ scores within each condition group. Scores ranged between 1 and 5.

Post‐hoc Bonferroni‐corrected simple main effects analyses showed that within the negative group stigma significantly increased from Pre [*M* = 1.54, *SD* = 0.37, 95% CI (1.41, 1.67)] to Post [*M* = 1.92, *SD* = 0.78, 95% CI (1.65, 2.20), *p* < .001]. However, no significant difference was identified within the positive group from Pre [*M* = 1.45, *SD* = 0.30, 95% CI (1.34, 1.55)] to Post [*M* = 1.35, *SD* = 0.37, 95% CI (1.22, 1.48), *p* = 1.000] or within the neutral group from Pre [*M* = 1.52, *SD* = 0.37, 95% CI (1.39, 1.65)] to Post [*M* = 1.62, *SD* = 0.57, 95% CI (1.43, 1.82), *p* = 1.000]. Pre and Post comparisons as a function of condition group are displayed in Figure 1. Therefore, stigma significantly increased from pre–post‐exposure in the negative stimuli condition. Comparing scores between groups at the Pre timepoint, no difference in stigma was identified between the negative and positive [*p* = 1.000] or neutral groups [*p* = 1.000], nor between the positive and neutral groups [*p* = 1.000]. Whereas comparing scores between groups at the Post timepoint, significantly higher scores were identified in the negative condition than in the positive condition [*p* = .002]. However, no difference in stigma scores was identified between Post ratings within the neutral and negative [*p* = .571] or positive groups [*p* = .923].

### Broad conceptualization of beauty

For conceptualizations of beauty, there was no significant main effect for Group [*F*(2, 100) = .93, *p* = .397, η^2^ = .022] or Time [*F*(1, 100) = .97, *p* = .326, η^2^ = .010] and no significant interaction effect [*F*(2, 100) = 2.98, *p* = .056, η^2^ = .018]. Therefore, no significant difference in conceptualizations of beauty was identified following exposure to any of the conditions.

### Body appreciation

For body appreciation, there was no significant main effect of Group [*F*(2, 100) = .40, *p* = .675, η^2^ = .008], but there was a significant main effect for Time [*F*(1, 100) = 16.06, *p* < .001, η^2^ = .138]. Additionally, there was no statistically significant interaction found between Group and Time [*F*(2, 100) = 1.58, *p* = .210, η^2^ = .031]. Examination of means displayed that overall body appreciation scores significantly increased from Pre [*M* = 3.28, *SD* = 0.85, 95% CI (3.12, 3.45)] to Post [*M* = 3.41, *SD* = 0.89, 95% CI (3.24, 3.59), *p* < .001], indicating that body appreciation increased across all conditions, with no significant difference between groups.

## DISCUSSION

The aim of the current study was to examine whether exposure to images depicting visible differences in different contexts (either positive, negative or neutral) could significantly impact individuals' levels of stigma towards individuals with visible differences and their own body appreciation and broad conceptualization of beauty. Overall, the study demonstrated statistically significant effects on individuals' levels of stigma towards visible difference and observers' own body appreciation. There were, however, no significant differences found regarding how respondents explicitly conceptualize beauty.

In line with previous research, participants' levels of stigma towards visible difference were shown to be significantly affected by the images they were shown. Firstly, respondents in the negative condition displayed significantly more stigmatizing attitudes after viewing photographs of individuals with visible differences as villains in popular films and television. This supports previous research findings that negative representations within television and film contribute to negative attitudes and stigma towards individuals with visible differences (Mantey, 2017; Rumsey & Harcourt, 2012; Sarwer et al., 2022). These attitudes likely give rise to the aversive behaviours exhibited by the public towards individuals with visible differences (e.g., staring, unwanted questions and comments) which can result in negative psychosocial outcomes and the internalization of stigma (Scambler, 2009; Spencer et al., 2016; Zhang & Haller, 2013). As a result, the findings of this study emphasize the damaging influence of negative portrayals of people with visible differences in film and provide empirical support for campaigns such as Changing Faces in their ‘I am not your villain’ campaign, calling for the film industry to stop using visible differences (e.g., scarring, skin conditions, limb loss) to portray villains.

On the other hand, in contrast to expectations, within the positive condition no significant difference was identified in appearance‐related stigma after participants viewed images of people with visible differences portrayed positively. While it was anticipated that exposure to positive representations of people with visible differences would result in parasocial contact leading to reduced stigma, the lack of positive effect indicates that the conditions for fostering parasocial contact were not met within the stimulus exposure. Previous research has found that repeated exposures over an extended period are often required to create a deep parasocial bond, which allows for the perspective taking required for stigma to be reduced (Bond, 2016, 2021). Thus, it is likely that within the present study, the single round of exposures to images with attached narratives was insufficient to create a parasocial bond between the participants and the individuals within the stimuli. In addition, the contrasting effects identified within the Mathews et al. (2023) study in which participant stigma was reduced after viewing a documentary depicting the lived experiences of individuals with scarring likely reflect the importance of representations including in‐depth experiential narratives for building parasocial relationships. People are more likely to relate to and identify with content depicting a more personal account of the lived experiences of individuals with visible differences, resulting in greater reductions of stigma. In line with this, Rasset et al. (2025) found that using five descriptive identity labels was necessary to reduce stigma towards individuals living with alcoholism and ‘rehumanise’ them. This further emphasizes the potential utility of social media as a medium for stigma reduction, as users are repeatedly exposed to content depicting the personal lives, aspects of identity, interests and experiences of those that they follow. Future research should seek to assess whether exposure to social media content from individuals with visible differences can foster parasocial contact and reduce stigmatizing attitudes.

Alternatively, it is possible that the high proportion of the sample reporting contact with persons with visible differences (see Table 1), resulted in participants being highly sensitized to visible difference, potentially nullifying any positive effect of the exposure. As direct and indirect contact with a stigmatized group is associated with reduced levels of stigma (e.g., Kilby & Mickelson, 2025; Pettigrew & Tropp, 2008), the already sensitized sample likely recorded low baseline stigma, which was not further reducible through the positive exposure. As such, a greater stigma‐reducing effect within the positive condition might have been identified with a less sensitized sample. However, as approximately one‐fifth of the UK population are estimated to have a visible difference (Changing Faces, 2019) and population‐level statistics on contact with individuals with visible differences are not available, it is unclear whether the present sample reflected above average levels of sensitization to visible difference. Consequently, future research specifically assessing the influence of familiarity or previous contact with people with visible differences on the stigma related effects of positive and negative representations of visible difference across a larger sample is needed to confirm the broader generalizability of the findings of the present study.

In relation to body appreciation, the present study found that viewing images of individuals with visible differences improved observers' attitudes towards their own bodies, regardless of whether the visible difference was portrayed positively, negatively or neutrally. This contrasts with expectations that the effect would only occur within the positive condition, as previous research has shown that exposure to positively framed images of people with diverse appearances can increase observers' body appreciation and satisfaction (Kilby & Mickelson, 2025; Stein et al., 2024). However, the pattern observed in the present study suggests that participants may instead have engaged in appearance‐based social comparison when viewing the stimuli. People have an inherent tendency to evaluate their own attributes and abilities by comparing themselves to others, including through appearance‐based social comparison (Thompson et al., 1999). Within the context of the current study, it is likely that the participants experienced downward social comparison—an adaptive process in which individuals enhance their self‐concept by selectively evaluating themselves against others perceived to be in a worse position (Wills, 1981). Appearance‐related downward social comparison has been found to be associated with increased body satisfaction and self‐esteem (Laker & Waller, 2022). In relation to downward social comparisons directed at people with visible differences specifically, Rumsey and Harcourt (2005) found that individuals without visible differences often derogate those with visible differences, perceiving them as ‘unattractive’ and ‘weak’. Such comparisons can enhance their own self‐perceptions and satisfaction with their appearance. This was demonstrated in a qualitative study conducted by Saeedinezhad et al. (2016), wherein one participant stated, ‘although my skin isn't perfect, it's so much better than my neighbour's skin, who has vitiligo’. Taken together, these findings suggest the possibility that the observed increase in body appreciation post‐exposure may be the result of participants engaging in appearance‐based downward social comparisons with the individuals with visible differences in the stimulus images, thereby enhancing their own appearance‐related self‐evaluations. Future research is required to specifically explore whether the effect is the result of downward social comparison, as if this is confirmed, it could be considered an undesirable spillover effect of positive representation which would require careful consideration by researchers and body‐positive influencers seeking to design anti‐stigma interventions and media campaigns. However, as this study did not measure the exact mechanism which resulted in the identified body appreciation effect, this latter point is made tentatively.

Contrary to the third hypothesis, conceptualizations of beauty did not change following exposure in any of three conditions. One possible explanation for this might be that a state outcome measure was not used. Rather than measuring characteristic patterns of thinking, feeling and behaving in a concrete situation at a specific moment in time (Matthews et al., 1999), this measure focused on characteristic patterns that generalize across similar situations, differ systematically between individuals and remain somewhat stable across time (Watson & Walker, 1996). This therefore makes it extremely difficult to change individuals' attitudes which have been reinforced and embedded from a very young age, especially when most people are already reluctant to change their attitudes regarding what the best approach is, or to shift their outlook and reframe their goals (Krüger et al., 2016). As stated previously, appearance‐related stigma can emerge in early childhood by the age of four years, whereby they already categorize people into disabled and nondisabled and favour the nondisabled (Parnell, 2021). Due to these conditioned biases, and the fact most people are highly resistant to persuasion—which is especially true when a short‐term attempt at changing a person's view is made—this may explain why no significant results were found. Lasting, meaningful attitude change occurs gradually, over the course of weeks, months and sometimes years (Briñol et al., 2015). Therefore, future researchers should consider using a state measure, within a longitudinal study, which aims to gradually increase broad conceptualizations of beauty. This might be in the form of following a specific social media account for a prolonged period of time.

### Limitations and future research

A key limitation of this study concerns the asymmetry of the valence of the manipulation between conditions. Specifically, while the manipulation targeted the context within which the visible difference was displayed (e.g., positive, negative or neutral), the positive condition displayed the visible difference in a positive light with descriptions of the individual's life and effects of the condition or injury, whereas negativity was operationalized through descriptions of the fictional villains' crimes, which did not specifically relate to their visible difference. As a result, the conditions may not be equivalent in the variable they valence, which must be considered when interpreting the findings.

Additionally, although the outcome measures used within this study have been used within previous research, some were also adapted from validated measures which do not allow for the contextualization of baseline scores against established normative values. Furthermore, despite this study demonstrating how an individual's stigma and body appreciation can be significantly changed after observing images of visible difference portrayed positively, it was not possible to examine whether the intervention caused real‐life changes in attitudes and behaviours. For example, in the work environment, during daily activities or at home. Given that this could have a wider impact on attitudes surrounding diversity of appearance in the general population, it would be beneficial for researchers to explore whether there were any broader, longer‐term impacts of the intervention.

Finally, as the study relied on explicit outcome measures, social desirability bias (SDB) might have influenced the results. Socially desirable responding involves participants answering questions in a manner that will be viewed favourably by others, which might not be reflective of their true views (Paulhus, 2002). Research involving explicit ratings of attitudes towards societally marginalized groups is particularly susceptible to SDB (e.g., Pazhoohi et al., 2021). As the positive and neutral conditions included exposure to images of real people with visible differences, SDB likely influenced participant responses (e.g., participants responding more positively than is reflected in their true attitudes) to a greater extent than in the negative condition which included images of fictional characters. This may explain some of the between‐condition variance in participant appearance‐based stigma scores identified in this study. To minimize the influence of SDB, future research should consider measuring and controlling for participant SDB or assessing implicit attitudes through an implicit associations test (IAT, Greenwald et al., 1998). However, it is important to note that recent research has critiqued the use of implicit measures and argued that explicit self‐report measures are more effective instruments for attitude research (Corneille & Gawronski, 2024; Corneille & Hütter, 2020).

## CONCLUSIONS

In summary, findings from the current study suggest that stigma towards visible difference can be easily negatively influenced, highlighting the importance of addressing the negative portrayal of visible difference within both the media and film industries. In contrast, reducing stigma through positive exposure was displayed to be more challenging, with implications for the design of stigma reduction interventions. Findings also suggest that body appreciation can be altered; however, the experimental exposure did not significantly increase broad conceptualizations of beauty. Given that broad conceptualizations are important components of appearance‐related interventions, researchers should therefore consider ways in which to increase this, for example by conducting a longitudinal study or by increasing discussions surrounding visible difference. Practically, the findings support campaigns that call for the film industry to stop portraying villains as having visible differences.

## AUTHOR CONTRIBUTIONS

**Harley Teagle:** Conceptualization; methodology; data curation; formal analysis; writing – original draft. **Maia Thornton:** Conceptualization; methodology; supervision; writing – review and editing. **Wylde M. C. Roberts‐Mills:** Formal analysis; writing – review and editing. **Ella Guest:** Conceptualization; methodology; supervision; writing – review and editing.

## CONFLICT OF INTEREST STATEMENT

The authors have no conflicting or competing interests to declare that are relevant to the content of this article.

## Data Availability

The data that support the findings of this study are available on request from the corresponding author. The data are not publicly available due to privacy or ethical restrictions.
